# Severe harm from others' drinking: A population‐based study on sex differences and the role of one's own drinking habits

**DOI:** 10.1111/dar.13202

**Published:** 2020-10-20

**Authors:** Erica Sundin, Maria Rosaria Galanti, Jonas Landberg, Mats Ramstedt

**Affiliations:** ^1^ Department of Clinical Neuroscience Karolinska Institutet Stockholm Sweden; ^2^ The Swedish Council for Information on Alcohol and Other Drugs Stockholm Sweden; ^3^ Department of Global Public Health Karolinska Institutet Stockholm Sweden; ^4^ Centre for Epidemiology and Community Health Stockholm County Council Stockholm Sweden; ^5^ Department of Public Health Sciences Stockholm University Stockholm Sweden

**Keywords:** alcohol, harm to others, severity, sex, interactions

## Abstract

**Introduction and Aims:**

Despite the fact that many studies have focused on harm from others' drinking, there is a lack of knowledge regarding severe forms of these harms. This study aimed to assess sex differences in the prevalence of severe harm from others' drinking and sex‐specific associations with one's own drinking.

**Design and Methods:**

The data originated from a Swedish cross‐sectional population survey (*n* = 15 576). Adjusted odds ratios of self‐reported experiences of severe harm (harmed ‘a lot’) from others' drinking were calculated using logistic regression models. Additive interactions were used to determine sex‐specific associations between own drinking and harm.

**Results:**

The past‐year prevalence of severe harm from known and unknown drinkers was higher among women (4.9% and 1.8%, respectively) than men (1.9% and 1.2%, respectively). Alcohol dependence predicted such harm for both sexes. No association with severe harm from known drinkers was found for male drinkers and binge drinkers, whereas female drinkers and binge drinkers reported more experiences of such harm. These differences indicated a supper‐additive interaction (RERI: 0.92–1.47) and signs of having alcohol dependence among women indicated an even higher interaction (RERI: 5.37).

**Discussion and Conclusions:**

Women suffer more frequently from severe harm from others' drinking. Men and women report different experiences of severe harm from known people's drinking conditioning on their drinking behaviour. Sex‐specific longitudinal studies are warranted to examine the relation between different behaviours and these harms. Whether these findings hold in settings with different drinking cultures and social norms should be explored.

## Introduction

Recent surveys on the epidemiology of alcohol‐related problems have increasingly emphasised the detrimental consequences for parties other than the drinker, that is, problems denoted as alcohol's harm to others (AHTO) [1, 2, 3]. These harms encompass a wide range of problems, such as being neglected by family members who drink, nuisances in public places and interpersonal violence. A thought‐provoking finding in these studies is that the prevalence of AHTO is generally higher among women than among men [4, 5, 6]. This is in contrast to alcohol's harm to the drinker, where 7.6% of the total disease burden in males and 2.2% in females is attributed to alcohol [7]. Another consistent result in these surveys is that AHTO is more common among heavy drinkers [8, 9], suggesting that drinking is a risk factor for both harm to the drinker and harm from others' drinking.

A limitation with previous research is that few studies have focused on severe harms from others' drinking, that is harms that are likely to have significant implications for health and social well‐being of the victim [10]. Consequently, little is known about the degree to which women and heavy drinkers have an elevated risk also for more severe cases of AHTO. One Australian study found sex differences in severe AHTO (measured as being harmed ‘a lot’ by someone else's drinking) that were similar to a general measure of AHTO including harms of different severity [11]. The extent of this result is generalisable to other countries and drinking cultures is not known, but it suggests that women tend to suffer more harm than men also for severe forms of AHTO.

In addition to a research gap in the extent own drinking and alcohol problems elevate the risk of severe AHTO, little is known of sex differences in this respect, that is if being a heavy drinker involves different risks of AHTO for men and women. More specifically, there is lack of studies assessing whether the combined effect of sex and own drinking behaviours exceeds the likelihood of harm from each factor considered individually [8]. Previous studies from Scandinavian countries offer support for sex‐specific patterns of harm when the results are stratified by sex, with a greater impact of own intoxication frequency among women than men on the risk of different types of AHTO [12, 13]. However, these studies do not answer the question of whether a certain level or pattern of drinking entails a higher risk of severe AHTO among women than among men.

The aims of the present study were to assess sex differences in the prevalence of severe harm from other people's drinking and to explore sex‐specific associations between one's own drinking habits (including own alcohol problems) and severe harm. Using data from a large Swedish population sample, the following research questions will be addressed:

1. Is the self‐reported prevalence of severe harm from other people's drinking higher among women than men?
2. To what extent is the association between own drinking habits and severe harm from other people's drinking modified by sex?

As AHTO may occur either due to known people's drinking (e.g. that of family members, friends or workmates) or due to the behaviour of unknown drinkers [1], the analyses will be conducted separately for these two different types of severe harm.

## Methods

### *Study design*

A cross‐sectional survey was conducted in Sweden between February and May 2013. The original sample included 27 000 individuals aged 17–84 years (thus, born 1929–1996) and randomly selected from the Swedish population register, which includes all residents in the country. Participants were asked to complete a paper‐and‐pencil questionnaire or an identical web‐based questionnaire. A total of three reminders were sent to non‐responders. A voucher of 100 SEK (approximately 10 Euro) was offered to the participants. In cases where respondents answered the questionnaire more than once, duplicates were removed and the first registered questionnaire was included in the data file. A total of 15 576 (57.7%) individuals completed the survey. The number of respondents unable to participate due to illness, emigration, language problems and death was 222, and 521 addresses were incorrect. The study was approved by the Central Ethical Review Board in Stockholm (reference number 2012/1740‐31/5).

### *Measures*

The respondent's own drinking habits were assessed as: (i) frequency of drinking during the past 12 months; and (ii) frequency of binge drinking during the past 12 months, defined as having approximately six units of alcohol [corresponding to one bottle of wine, five glasses of spirits (4 centilitres), four cans of regular strength beer or six cans of ‘folk’ beer containing 2.8–3.5% alcohol] or more on the same drinking occasion. For both variables, participants were grouped into the following categories: non‐(binge) drinkers, up to three times a month and one time or more a week.

Alcohol dependence (yes, no) was assessed by study‐specific questions based on the Swedish version of the Mini International Neuropsychiatric Interview version 6 [14], which covers Diagnostic and Statistical Manual of Mental Disorders, fourth edition (DSM‐IV) alcohol dependence symptoms (see Table SS1, Supporting Information, for wording of items). To be categorised as alcohol dependent, three or more of the seven DSM‐IV symptoms [15] needed to be reported. The seven symptoms of alcohol dependence are characterised by loss of control over drinking, increased tolerance of alcohol and withdrawal upon cutting down alcohol intake.

The study‐specific questions on self‐reported AHTO were derived from the World Health Organization–ThaiHealth protocol [16]. Before assessing perceived severe harm, the respondents were asked two filter questions about harm from known people's drinking in general, including a question whether the respondent had someone in their life who was a heavy drinker, (Filter question A1–A2, Table 1) and one filter question about harm from strangers' drinking in general (Filter question B1, Table 1). For those answering affirmatively to these filter questions, perceived severe harm was assessed for harm from known people's and strangers' drinking separately, by asking: ‘Overall, how much has the person's/persons’ drinking affected you negatively?’ with the response alternatives ‘a lot’ and ‘a little’ (Question A3 and B2, Table 1). All questions referred to the past 12 months. For the purpose of the present analysis, responses were dichotomised into ‘reports being harmed a lot’ versus any other response alternative. A previous study from Australia showed that people who reported being harmed ‘a lot’ by someone else's drinking also on average rated that harm using a score of around 8 on a linear 1‐to‐10 scale [17].

**Table 1:** Wording of items

Wording of item	Response alternatives
*Harmed a lot by known people's drinking*	
A1, filter question:	‘Yes’, ‘No’
‘During the last 12 months, has there been someone in your life that you consider to have been drinking too much alcohol (it can be on a regular basis or occasionally)?’ Examples of people in your life: family, ex‐partner, friends or colleagues.	
A2, if yes, filter question:	‘Yes’, ‘No’
‘Has that person's/those persons’ drinking affected you negatively in some way?’	
A3, if yes:	‘A little’, ‘A lot’
‘Overall, how much has the person's/persons’ drinking affected you negatively?’	
*Harmed a lot by strangers' drinking*	
B1, filter question:	‘Yes’, ‘No’
Have you, during the last 12 months, been negatively affected by the drinking of strangers or people you do not know very well? For example, not been able to sleep, been insulted or afraid, been harmed or assaulted, have had property damaged.	
B2, if yes:	‘A little’, ‘A lot’
Overall, how much has the person's/persons' drinking affected you negatively?	

### *Statistical analysis*

Descriptive information about the study sample is presented as proportions, and differences in the distribution between men and women were examined using Pearson's χ^2^.

We estimated the self‐reported past‐year prevalence of harm and corresponding 95% confidence intervals (CI) for men and women overall as well as within subgroups for different drinking behaviour. The past‐year prevalence of severe harm was considered as differing between groups if the 95% CIs did not overlap.

The association between own drinking habits, sex and severe harm were estimated by calculating odds ratios (OR) and corresponding 95% CIs using binary logistic regression, after adjustment for covariates that could represent potential confounders; age in intervals (17–35, 36–64, 65–84 years) [11], level of education [low (compulsory school), medium (upper secondary school), high (tertiary education)] [5], partner status (not living with a partner, living with a partner) and living in an urban area (no, yes) [9].

First, single effects were estimated for sex and drinking habits. The single effect refers to the estimate of sex without stratifying on drinking habits, or the reverse. Next, to assess whether the association between own drinking habits and severe harm from others' drinking was modified by sex, we estimated joint effects for sex and each indicator of own drinking habits (drinking frequency, frequency of binge drinking, alcohol dependence) separately using a common reference category for the two factors. The references categories were as follows: non‐drinking men, non‐binge drinking men and men without signs of alcohol dependence. Separate models for every single exposure and joint exposures were fitted.

Interactions on the additive scale were then calculated using Rothman's [18] formula for relative excess risk due to interaction (RERI) (RERI=OR_11_ − OR_10_ − OR_01_ + 1). This measure estimates whether the combined OR of the two factors together (OR_11_) is larger (or smaller) than the sum of the ORs of the two factors considered individually (OR_10_, OR_01_). In addition, the attributable proportion (AP) to interaction (RERI/OR_11_) was calculated to estimate the proportion of the combined effect that is due to interaction. CIs for RERI were calculated using the delta method [19] as recommended by VanderWeel and Knol [20].

In order to assess if the included joint effect variables statistically and significantly contributed with improvements of the model fit, Wald tests were calculated for different categories of each joint variable of sex and own drinking habits. As an example, the Wald test was calculated for improvement of model fit when including the categories of men and women, respectively, drinking weekly in comparison to the reference category of non‐drinking men.

The data were weighted to reproduce the sex and age composition of the Swedish population in the 2013 census. Data analyses were carried out using the Stata Statistical Software version 16 [21]. Respondents with missing values in the relevant variables included in the analyses were excluded. Missing information was below 5.5% for all variables.

## Results

### *Characteristics of the study sample*

The characteristics of the analytical sample are presented in Table 2, separated by sex. A larger proportion of women compared to men belonged to the oldest age category (65–84 years) and to the category with the highest level of education. No difference was found between men and women regarding living conditions. Drinking habits and alcohol dependence differed significantly by sex, with men drinking more frequently, more heavily and more often showing characteristics compatible with alcohol dependence. Experiencing any harm from other people's drinking, regardless of the severity of harm, was more frequent among women, particularly concerning harm from persons known to the respondent. Approximately one‐in‐five women (19.4%) compared to one‐in‐ten men (10.8%) reported harm from a known drinker, while the corresponding proportions for harm from strangers' drinking were 10.7% and 9.0%.

**Table 2:** Analytical sample characteristics

	Men	Women		All
*n*	7797	7779		15 576
	% (*n*)	% (*n*)	*P*‐values	*% (n)*
*Age groups, years*			0.002	
17–35	30.5 (2364)	29.8 (2312)		30.1 (4676)
36–64	49.7 (3856)	48.0 (3716)		48.8 (7572)
65–84	19.9 (1544)	22.2 (1720)		21.0 (3263)
*Level of education*			<0.001	
Low	21.4 (1593)	19.8 (1459)		20.6 (3051)
Medium	42.3 (3153)	34.1 (2508)		38.2 (5661)
High	36.3 (2702)	46.1 (3387)		41.1 (6089)
*Partner status*			0.895	
Not living with a partner	37.2 (2903)	37.3 (2904)		37.3 (5807)
Living with a partner	62.8 (4894)	62.7 (4875)		62.7 (9769)
*Living in an urban area*			0.077	
No	83.2 (6484)	82.1 (6378)		82.7 (12862)
Yes	16.8 (1307)	17.9 (1390)		17.3 (2697)
*Drinking frequency*			<0.001	
Non‐drinkers	9.7 (750)	13.3 (1028)		11.5 (1778)
Up to 3 times a month	36.8 (2841)	47.0 (3620)		41.9 (6461)
1 time or more a week	53.5 (4133)	39.7 (3056)		46.6 (7189)
*Frequency of binge drinking*			<0.001	
Non‐binge drinkers	24.3 (1868)	45.6 (3482)		34.9 (5350)
Up to 3 times a month	56.1 (4313)	46.2 (3533)		51.2 (7846)
1 time or more a week	19.5 (1501)	8.2 (627)		13.9 (2128)
*Alcohol dependence*			<0.001	
No	94.5 (7370)	97.0 (7547)		95.8 (14917)
Yes	5.5 (427)	3.0 (232)		4.2 (659)
*Reported harm from known people's drinking (regardless of severity)*			<0.001	
No	89.2 (6777)	80.6 (6049)		85.0 (12827)
Yes	10.8 (820)	19.4 (1452)		15.0 (2272)
*Reported harm from stranger's drinking (regardless of severity)*			0.001	
No	91.0 (6866)	89.3 (6694)		90.1 (13560)
Yes	9.0 (682)	10.7 (806)		9.9 (1488)

*Note*: the denominator includes all participants excluding missing cases. Weighted percentages and numbers.

### *Sex differences in the prevalence of severe harm from other people's drinking*

The past‐year prevalence of severe AHTO was higher among women than men, in particular regarding known drinkers (4.9% and 1.9%, respectively, Table 3) but also for strangers' drinking (1.8% and 1.2%, respectively). Higher estimates of harm from known people's drinking were found among women than among men in most subgroups defined by the frequency of drinking, binge drinking and alcohol dependence. However, among non‐drinkers no sex difference was found. The prevalence of severe harm from strangers' drinking did not differ significantly between men and women in any subgroup.

**Table 3:** Past‐year prevalence (%) of severe harm (harmed ‘a lot’) from known people's and strangers' drinking according to own drinking patterns and sex

	Harmed ‘a lot’ by known people's drinking				Harmed ‘a lot’ by strangers' drinking			
	Men		Women		Men		Women	
	n/N	% (95% CI)	n/N	% (95% CI)	n/N	% (95% CI)	n/N	% (95% CI)
All	145/7569	1.9 (1.6, 2.3)	366/7433	4.9 (4.5, 5.4)	88/7532	1.2 (0.9, 1.5)	134/7478	1.8 (1.5, 2.1)
*Drinking frequency*								
Non‐drinkers	16/716	2.3 (1.3, 3.9)	26/967	2.7 (1.8, 3.9)	16/710	2.2 (1.3, 3.9)	23/948	2.4 (1.6, 3.6)
Up to 3 times a month	56/2772	2.0 (1.5, 2.7)	202/3475	5.8 (5.1, 6.6)	32/2762	1.2 (0.8, 1.7)	73/3521	2.1 (1.6, 2.6)
1 time or more a week	72/4035	1.8 (1.4, 2.3)	135/2947	4.6 (3.9, 5.4)	39/4025	1.0 (0.7, 1.4)	37/2970	1.2 (0.9, 1.7)
*Frequency of binge drinking*								
Non‐binge drinkers	29/1804	1.6 (1.1, 2.4)	118/3330	3.5 (3.0, 4.2)	27/1800	1.5 (1.0, 2.3)	49/3341	1.5 (1.1, 1.9)
Up to 3 times a month	75/4218	1.8 (1.4, 2.3)	200/3417	5.9 (5.1, 6.7)	45/4203	1.1 (0.8, 1.5)	66/3443	1.9 (1.5, 2.5)
1 time or more a week	41/1469	2.8 (2.0, 3.9)	44/594	7.4 (5.5, 9.8)	16/1456	1.1 (0.6, 1.8)	17/611	2.8 (1.7, 4.5)
*Alcohol dependence*								
No	116/7154	1.6 (1.3, 2.0)	326/7216	4.5 (4.1, 5.0)	72/7114	1.0 (0.8, 1.3)	118/7256	1.6 (1.4, 1.9)
Yes	29/415	6.9 (4.6, 10.3)	41/217	18.7 (13.9, 24.7)	17/418	4.0 (2.4, 6.8)	16/222	7.2 (4.3, 11.6)

*Note*: the denominator includes all cases excluding missing cases. Weighted percentages and numbers. CI, confidence interval.

### *Single and joint associations of own drinking habits and alcohol dependence with severe harm*

The adjusted single associations resembled those in the bivariate analyses and show that severe AHTO is more frequent among women than men (Table 4). In general, own drinking frequency and binge drinking were associated with increased odds of severe harm from known people's drinking, although the association was only statistically significant for those drinking up to three times a month compared to non‐drinkers. In contrast, frequency of drinking and binge drinking were associated with decreased odds of severe harm from strangers' drinking. However, the association was only statistically significant for drinking frequency. Moreover, persons with signs of having an alcohol dependence had increased odds of being severely harmed by both known people's and strangers' drinking.

**Table 4:** Adjusted^a^ odds ratios (single and joint effects using a common reference group) and measures of additive interactions (RERI) between sex and drinking habits of severe harm (harmed ‘a lot’) from known people's and strangers' drinking

		Harmed ‘a lot’ from known people's drinking						Harmed ‘a lot’ from strangers' drinking				
		Joint effect						Joint effect				
	Single effect	Men	Women		Additive interaction		Single effect	Men	Women		Additive interaction	
	OR (95% CI)	OR (95% CI)	OR (95% CI)	Wald test^b^	RERI (95% CI)	AP	OR (95% CI)	OR (95% CI)	OR (95% CI)	Wald test^b^	RERI (95% CI)	AP
*Sex* ^c^												
Men	1.00	N/A	N/A	N/A	N/A	N/A	1.00	N/A	N/A	N/A	N/A	N/A
Women	2.82*** (2.26, 3.52)	N/A	N/A	N/A	N/A	N/A	1.51** (1.11, 2.06)	N/A	N/A	N/A	N/A	N/A
*Drinking frequency* ^c^												
Non‐drinkers	1.00	1.00	1.29 (0.65, 2.54)	N/A	N/A	N/A	1.00	1.00	1.11 (0.53, 2.33)	N/A	N/A	N/A
Up to 3 times/month	1.47* (1.03, 2.09)	0.79 (0.41, 1.50)	2.55** (1.42, 4.57)	*P* < 0.001	1.47** (0.74, 2.21)	0.58	0.65* (0.42, 1.00)	0.47* (0.23, 0.99)	0.87 (0.45, 1.69)	*P* < 0.05	0.28 (−0.43, 0.99)	0.33
1 time a week or more	1.21 (0.84, 1.75)	0.83 (0.44, 1.56)	2.39** (1.31, 4.34)	*P* < 0.001	1.27** (0.54, 1.99)	0.53	0.47** (0.30, 0.75)	0.46* (0.22, 0.95)	0.57 (0.28, 1.16)	*P* = 0.140	−0.01 (−0.82, 0.80)	−0.01
*Frequency of binge drinking* ^c^												
Non‐binge drinkers	1.00	1.00	2.35*** (1.48, 3.72)	N/A	N/A	N/A	1.00	1.00	1.06 (0.61, 1.84)	N/A	N/A	N/A
Up to 3 times/month	1.04 (0.83, 1.30)	0.92 (0.56, 1.53)	3.19*** (2.02, 5.02)	*P* < 0.001	0.92** (0.21, 1.63)	0.29	0.76 (0.54, 1.08)	0.61 (0.34, 1.11)	1.00 (0.57, 1.73)	*P* = 0.057	0.32 (−0.24, 0.88)	0.32
1 time or more a week	1.25 (0.93, 1.68)	1.53 (0.88, 2.67)	4.28*** (2.51, 7.28)	*P* < 0.001	1.40 (−0.16, 2.95)	0.33	0.89 (0.56, 1.41)	0.66 (0.32, 1.36)	1.52 (0.76, 3.05)	*P* = 0.140	0.80 (−0.12, 1.72)	0.53
*Alcohol dependence* ^c^												
No	1.00	1.00	3.05*** (2.39, 3.90)	N/A	N/A	N/A	1.00	1.00	1.57** (1.12, 2.21)	N/A	N/A	N/A
Yes	3.23*** (2.39, 4.36)	3.99*** (2.44, 6.50)	11.41*** (7.40, 17.61)	*P* < 0.001	5.37* (0.70, 10.05)	0.47	2.86*** (1.84, 4.44)	3.03** (1.60, 5.75)	4.68*** (2.48, 8.83)	*P* < 0.001	1.08 (−2.07, 4.23)	0.23

*P < 0.05, ** *P* < 0.01, *** *P* < 0.001.

^a^ Adjusted for age groups, level of education, partner status and living in an urban area.

^b^ Non‐weighted.

^c^ Separate models.

AP, attributable proportion; CI, confidence interval; N/A, not applicable; OR, odds ratio; RERI, relative excess risk due to interaction.

When sex and own drinking habits were considered jointly in the adjusted models, the results showed that the association between one's own drinking habits and severe harm from known people's drinking was modified by sex (Table 4). That is, compared to the reference groups male non‐drinkers and male non‐binge drinkers, no statistically significant association was found for male drinkers and binge drinkers. However, compared to the same reference groups, female drinkers and binge drinkers reported more experiences of severe harm from known people's drinking, with ORs ranging from 2.39 to 4.28. These results corresponded to superadditive interactions, with RERI estimates between 0.92 and 1.47, and AP estimates between 0.29 and 0.58. More specifically, the RERI of 1.47 implies that, compared to non‐drinking men, the OR of the double‐exposed group, women that drink one time a week or more, is 1.47 times larger than the sum of the ORs from the two single exposed groups (non‐drinking women and men that drink one time a week or more, respectively). The corresponding AP of 0.58 implies that 58% of the OR in the double‐exposed group is attributable to the interaction between own drinking habits and sex. In addition, the Wald tests of each category of the frequency of drinking and binge drinking variables in regards to severe harm from known drinkers indicated improvements of the models' fit (*P* < 0.001).

Conversely, for severe harm from strangers' drinking, we found no joint effect between own drinking habits and sex. Still, it should be noted that male drinkers had decreased odds of being severely harmed by strangers' drinking compared to male non‐drinkers.

Relative to men without alcohol dependence, women with signs of alcohol dependence had ORs of 11.41 and 4.68 for severe harm from known people's drinking and strangers' drinking, respectively. This corresponded to a statistically significant superadditive interaction of RERI 5.37 and AP of 47% for harm from known people's drinking, while no statistically significant RERI estimate was found for harm from strangers' drinking.

## Discussion

The aim of the present study was to assess sex differences in severe harm from others' drinking, as earlier studies have only used broader measures of AHTO also including less severe harms. Sex differences were assessed in terms of prevalence and the extent own drinking habits and problems are associated with this form of harm.

The prevalence of severe AHTO was significantly higher among women than among men, both regarding harm from known drinkers and from strangers' drinking. This sex difference was more pronounced for severe harm from known people's drinking, which was reported more than twice as often among women, whereas the prevalence of harm from strangers' drinking was about 50% higher.

Higher estimates of AHTO among women are in accordance with previous studies where severe and less severe harm have been mixed in the same measure [4, 8, 13]. The present findings, thus, add to the literature by demonstrating an elevated risk for women also regarding severe AHTO, that is harms likely to have a considerable impact on the victim's health and social wellbeing [10]. The most severe types of harm from others' drinking rated by alcohol researchers and policy experts in a key informant survey were physical, financial, practical and severe emotional harm [22].

One possible explanation for the higher prevalence of severe AHTO among women is that women are more aware of others' behaviour than men and, therefore, report problems more frequently in surveys. This interpretation is supported by studies based on register data showing that the same proportions of boys and girls have a parent with an alcohol‐related hospitalisation [23], whereas more women report living with a heavy drinker during their childhood in surveys [24]. This is also supported by a Finnish study conducted among relatives of problem drinkers [25] showing that women more often cared for the drinker, while denial was more common among men. In addition to sex differences in the perception of problems, however, it should be noted that most AHTO among women is determined by a close male person's drinking [26] and that men more often are heavy drinkers with a higher risk of causing harm to other people.

We also found a statistically significant joint effect of own alcohol habits and sex in relation to severe harm from known people's drinking, with a higher risk of harm associated with own drinking, binge drinking and signs of having alcohol dependence among women. Conversely, in relation to severe harm from stranger's drinking, no association between own drinking habits and such harm was found among women.

The positive association between own drinking and severe AHTO from known people among women is in line with previous studies based on data from the International Gender and Alcohol's Harm to Others project. One study from this project found that women who drink excessively are more likely to have a heavy‐drinking partner [27], with a resultant increased risk of being exposed to harmful behaviour from the male partner. The present results complement this knowledge by revealing that the likelihood of reporting severe harm from known people's drinking in one's nearby surroundings may not only be confined to women's partners, as severe harm in this study was not restricted to harm from partners.

Previous studies from Scandinavian countries offer support for sex‐specific patterns of harm in terms of own drinking habits. For instance, a Swedish population study based on data from 2002 showed that the association between own drinking habits and experiencing a minimum of two of the four specific types of AHTO was stronger among women than men. In the study, Hradilova Selin [12] revealed that the likelihood of harm increased with frequency of binge drinking among women, whereas no association was found among men. Similarly, based on a Norwegian population sample, Rossow and Hauge [13] found that the association between intoxication frequency and harms from others' drinking was stronger for women. Furthermore, recent data from outside the Scandinavian countries also showed the heightened risk for others' drinking‐related physical aggression among heavy drinking women [28].

One possible explanation for this sex difference is that, while under the influence of alcohol, women are more susceptible and at a higher risk of victimisation than men are. It was not possible to address this hypothesis in the present study, but previous studies suggest that intoxicated women are considered by others to be more vulnerable and more sexually available [29, 30].

It should be noted that, in relation to severe harm from strangers*'* drinking, own drinking frequency was negatively associated with harm, although in the joint effect models, the results indicated that this only was statistically significant for male drinkers and not among females. Explanations for this finding are so far speculative; it may be related to higher tolerance towards strangers' alcohol‐related misbehaviour with a higher own drinking frequency, or to a tendency to attribute a strangers' problem behaviour to other causes than alcohol. In fact, an Australian study showed decreased support for policies controlling hazardous alcohol‐behaviour among men experiencing abuse from others' drinking [31]. This result indirectly supports the explanation of a decreased likelihood to perceive serious harms among men harmed from others' drinking. Future studies are needed to explore this topic further.

Having signs of alcohol dependence was associated with an elevated risk of severe harm from others' drinking among both men and women. This may be explained by the fact that these individuals spend a lot of time in social contexts where alcohol problems are a shared experience, and likewise the occurrence of harm. There are no data in the current study to confirm this notion but a previous study showed that individuals' drinking increases with the number of heavy drinkers in their social networks [32]. It should further be noted that one of the criteria for dependence in DSM‐IV includes the influence of the individual's drinking on their social networks [15].

### *Strengths and limitations*

The major strength of the current study is the large sample size, which allows for a focus on the less prevalent severe forms of AHTO, but those entailing substantial risks for the health and the social well‐being of those exposed [10]. It should also be noted that the response rate of 58% is high, and other studies based on this survey data suggest that people with experiences of AHTO are well represented in the sample [33].

There are also some limitations that need to be acknowledged, such as the cross‐sectional design implying limited possibilities to draw any conclusions of causality. Another limitation is the broad definition of ‘severe’ harm which does not include information on types of harms and how often they have occurred.

## Conclusions

Women in Sweden suffer from severe harm from others' drinking to a higher extent than men and have a higher susceptibility to be harmed from known people's drinking in relation to own drinking behaviour. Severe alcohol problems such as alcohol dependence increase the risk of being severely harmed by others' drinking for both men and women.

## Supporting information


**Table S1.** Wording of study‐specific items of alcohol dependence and response alternatives.
