# Supplementary material for: Severe harm from others' drinking: A population‐based study on sex differences and the role of one's own drinking habits
Source: Drug Alcohol Rev. 2020 Oct 20;40(2):263–71. doi: 10.1111/dar.13202 (PMC7894316; doi:10.1111/dar.13202)
Supplement: Supplementary file 1 — Table S1. Wording of study‐specific items of alcohol dependence and response alternatives. [file DAR-40-263-s001.docx]

**Supplementary Table 1.** Wording of study-specific items of alcohol dependence and response alternatives.

| **Wording of item** | **Response alternatives** |
| --- | --- |
| During the past 12 months…: |  |
| …did you drink more in order to get the same effect that you got when you first started drinking? | ‘Yes’, ‘No’ |
| …has it happened that your hands shake, you start sweating or feel agitated when you cut down on drinking? | ‘Yes’, ‘No’ |
| …during the times when you drank alcohol, did you end up drinking more than you planned when you started? | ‘Yes’, ‘No’ |
| …have you tried to reduce or stop drinking alcohol but failed? | ‘Yes’, ‘No’ |
| …on the days that you drank, did you spend substantial time obtaining alcohol, drinking, or recovering from the effects of alcohol? | ‘Yes’, ‘No’ |
| …did you spend less time working, enjoying hobbies, or being with others because of your drinking? | ‘Yes’, ‘No’ |
| …have you continued to drink even though you knew that the drinking caused you health or mental problems? | ‘Yes’, ‘No’ |
